# Functional Relationships Between Wrist Joint Morphology and Ulnar Deviation in Strepsirrhine Primates

**DOI:** 10.1002/ajpa.70096

**Published:** 2025-07-17

**Authors:** Pierre Lemelin

**Affiliations:** ^1^ Division of Anatomy, Department of Surgery, Faculty of Medicine and Dentistry College of Health Sciences, University of Alberta Edmonton Alberta Canada

**Keywords:** carpals, hand, lemurs, locomotion, lorises

## Abstract

**Objectives:**

Ulnar deviation is a fundamental hand movement reflecting different positional behaviors that characterize primates and other arboreal mammals. Few experimental data exist on the relationship between wrist joint morphology and ulnar deviation of the hand in living primates. This study tests functional relationships between carpal joint anatomy and the degree of ulnar deviation for eight strepsirrhine species representing major locomotor groups.

**Methods:**

Passive ranges of ulnar deviation were measured from radiographs of the hand of 25 anesthetized subjects using a motion analysis software. Position of the carpal elements was also compared in two different anatomical positions (i.e., neutral and maximal ulnar deviation).

**Results:**

On average, the hand of pronograde quadrupeds (*Cheirogaleus*, *Eulemur*, *Hapalemur*, *Lemur*, and *Varecia*) showed ulnar deviation angles ranging between 31° and 50°. Compared to pronograde quadrupeds, ulnar deviation of the hand was greater (but not statistically significant) in the vertical clinging *Propithecus* (49° to 59°) and greatest (and statistically significant at *p* < 0.01) in slow climbing *Loris* and *Nycticebus* (64° to 95°).

**Conclusions:**

These differences in ulnar deviation of the hand closely match reported differences in proximal carpal and midcarpal joint shape in strepsirrhine primates. In pronograde quadrupedal and vertical clinging lemurs, ulnar deviation takes place mainly at the midcarpal joint, with some movement of the scaphoid and lunate at the proximal carpal joint of *Propithecus*. In slow climbing lorises, ulnar deviation of the hand is accompanied by equally notable movements of the carpals at both proximal carpal and midcarpal joints.

## 1 Introduction

The carpal bones are interconnected by complex joint surfaces that promote or restrict hand movements in different anatomical planes (Kauer and Landsmeer 1981). Among primates, variation in wrist joint morphology has been linked functionally to differences in hand position and mobility during positional behavior, including the degree of ulnar deviation (Daver et al. 2012; Hamrick 1996a, 1996b, 1996c; Jouffroy and Medina 2002). Ulnar deviation (i.e., displacement of the hand toward the ulnar side of the forearm) is a fundamental aspect of hand movement during the support phase of many locomotor behaviors observed in primates and other arboreal mammals (e.g., Cartmill and Milton 1977; Conroy and Fleagle 1972; Jouffroy and Medina 2002; Kivell 2016; Lemelin and Schmitt 1998; Lewis 1969; Mendel 1979; Neufuss et al. 2017; Orr et al. 2023; Preuschoft et al. 1993; Sarmiento 1988; Yalden 1972). Increased mobility of the hand, including ulnar deviation, is promoted by a series of morphological features at the proximal carpal and midcarpal joints. Proximally, the articular contribution of the lunate and radioulnar curvature of the joint are greater in *Hylobates* and *Pongo* compared to African apes (Jenkins and Fleagle 1975; Orr et al. 2023). This results in a ball‐and‐socket proximal carpal joint, which is associated with greater ulnar deviation of the hand in these highly suspensory primates compared to African apes and most cercopithecoids (Orr et al. 2023; Sarmiento 1988; Tuttle 1969a, 1969b). Ulnar deviation may also be promoted by the reduction of the ulnar styloid process and its isolation from the triquetrum and pisiform (Parsons 1899; Wood Jones 1942; O'Connor 1975), although the functional significance and evolution of this wrist feature for locomotor behavior remain contentious (Cartmill and Milton 1977; Conroy and Fleagle 1972; Jouffroy and Medina 2002; Kivell 2016; Lewis 1969, 1974, 1985, 1989; Orr et al. 2023). More distally, the articular surface of the hamate for the triquetrum of pronograde primates, especially terrestrial quadrupeds, is broader, oriented more proximally (i.e., radioulnar orientation), and bears a prominent “spiral” facet, which all promote stability and weight‐bearing abilities at the midcarpal joint when the hand is pronated and extended (Beard et al. 1986; Fleagle 1977; Fleagle and Meldrum 1988; Godinot and Beard 1991; Hamrick 1996a, 1996b, 1996c; Jenkins and Fleagle 1975; Kivell 2016; Lemelin et al. 2008; Orr et al. 2023; Richmond 2006; Richmond et al. 2001). In more orthograde primates such as clinging, climbing, and suspensory taxa, the same articular surface of the proximal hamate is oriented more laterally or proximodistally (i.e., proximal articular surface of the hamate forms a steeper slope with the distal articular surface of the triquetrum) and, together with the capitate, forms a bulbous knob with a greater radius of curvature that allows enhanced movement in different planes at the midcarpal joint, including greater ulnar deviation of the hand (Fleagle and Meldrum 1988; Hamrick 1996a, 1996b, 1996c; Hamrick et al. 2000; Jenkins 1981; Jenkins and Fleagle 1975; Kivell 2016; Orr et al. 2023; Sarmiento 1988).

**FIGURE 1:**
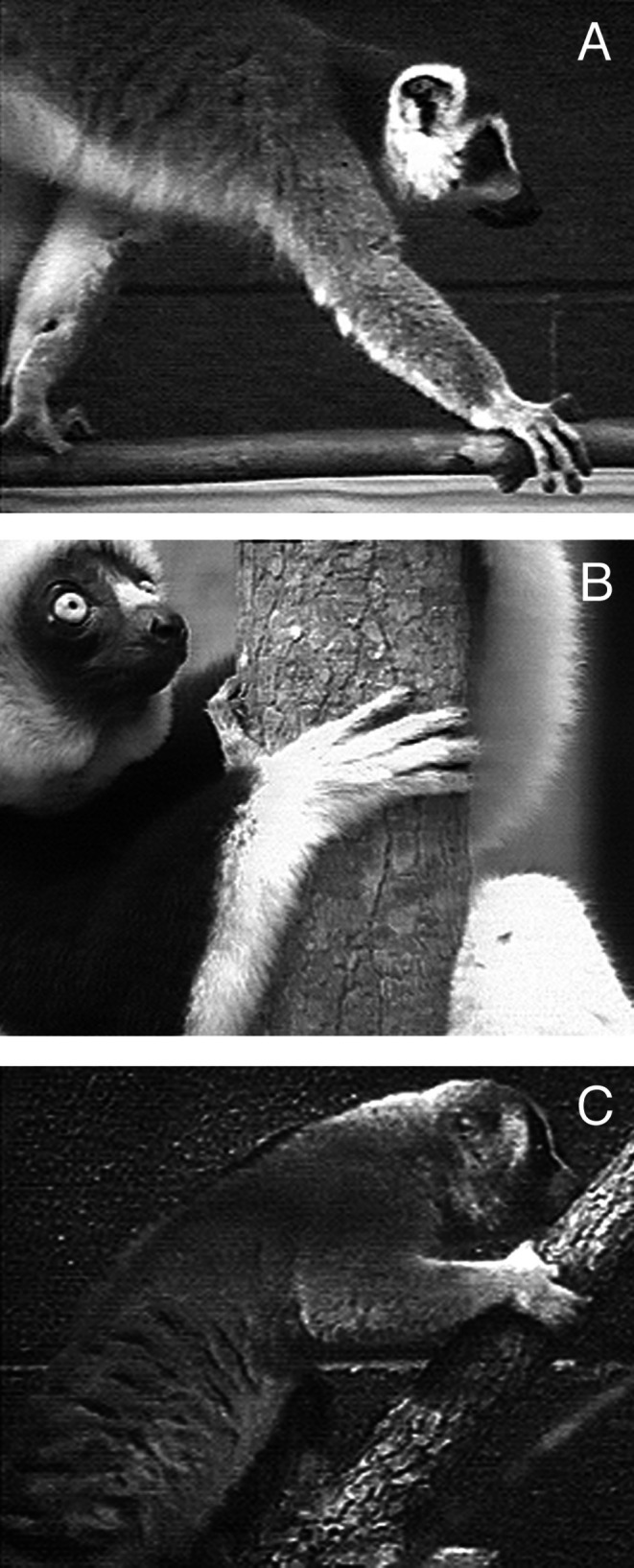
Hand position during positional behavior in three strepsirrhine primates. In pronograde quadrupeds like *Lemur catta* (A), the hand is held in a more neutral position with the substrate usually gripped between the second and third digits or along the second digit of the hand. The hand of vertical clingers like *Propithecus coquereli* (B) and slow climbers like *Nycticebus coucang* (C) shows more ulnar deviation, and the substrate is usually gripped between the first and second digits or along the second digit.

**FIGURE 2:**
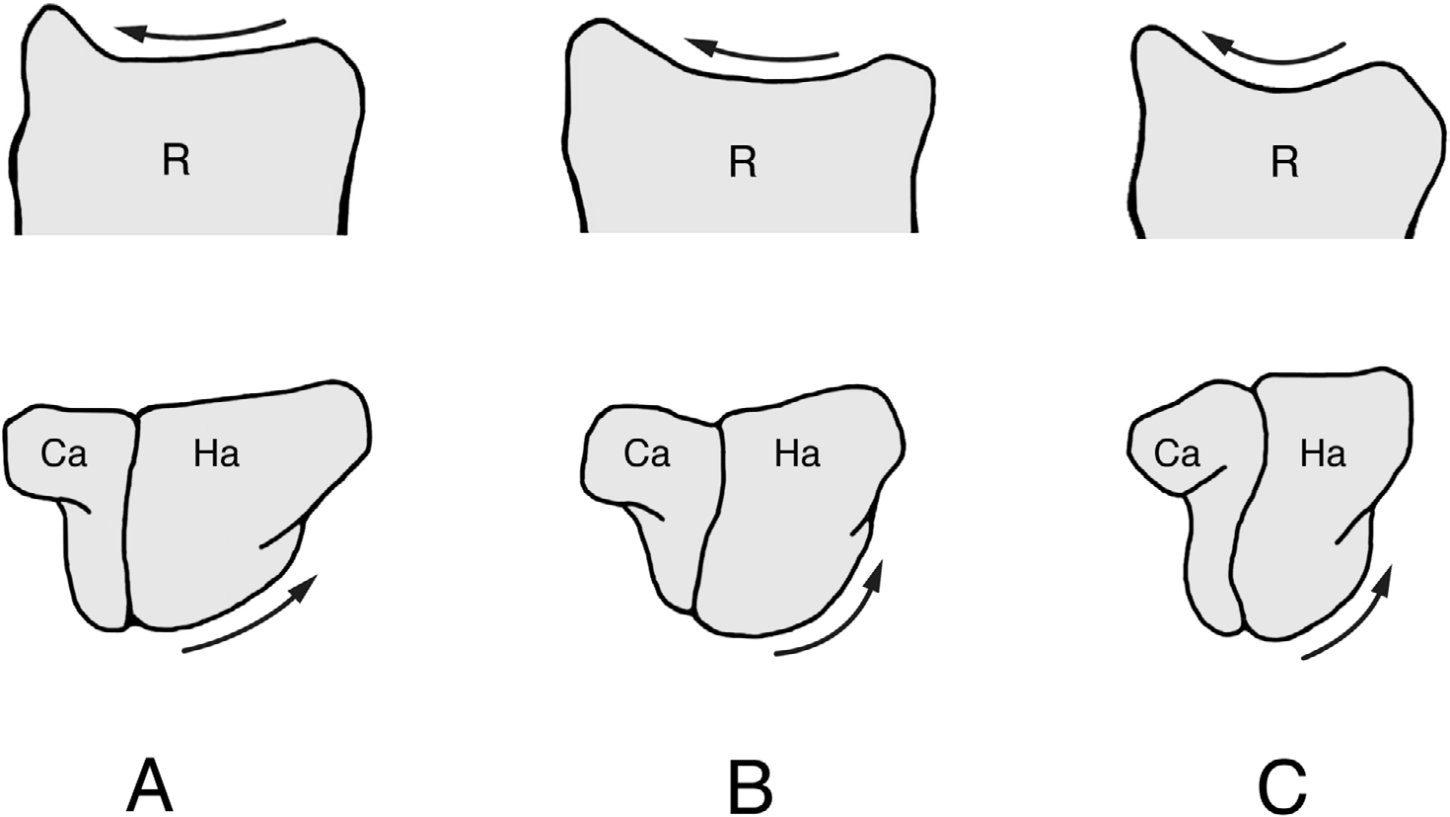
Transverse view of the distal radius (R) and dorsal view of the capitate (Ca) and hamate (Ha) of a pronograde quadruped ( *Eulemur fulvus* ) (A), a vertical clinger ( *Indri indri* on top and *Avahi laniger* on bottom) (B), and a slow climber ( *Nycticebus coucang* ) (C). On top, note the greater articular curvature of the distal radius of the slow climber compared to other taxa (arrows). At the bottom, note the progressively steeper sloping of the articular surface of the proximal hamate from the pronograde quadruped, vertical clinger to slow climber (arrows). Redrawn from Hamrick (1996a).

The hand of strepsirrhine primates shows considerable variation in positioning during locomotion and posture. In pronograde quadrupeds (e.g., cheirogaleids and lemurids), the hand is held in a more neutral position during the support phase of quadrupedal walking, with a branch being grasped along the axis of the second or third digit, or between the second and third digits (Bishop 1964; Hamrick 1996a, 1996b; Lemelin 1996; Lemelin and Schmitt 1998; Figure 1A). In vertical clinging indriids, the hand shows more ulnar deviation, with a branch being grasped between the first and second digits (Bishop 1964; Hamrick 1996a, 1996b; Lemelin 1996; Figure 1B). Ulnar deviation is most pronounced in slowclimbing lorises, allowing the forearm to be parallel with the substrate at touchdown of the support phase of walking and climbing (Hamrick 1996a; Jouffroy et al. 1983; Lemelin 1996; Sarmiento 1988; Figure 1C). In a series of influential contributions, Hamrick (1996a, 1996b, 1996c) documented quantitative differences in articular morphology of the proximal carpal and midcarpal joints of strepsirrhine primates that parallel these behavioral differences in ulnar deviation of the hand. Hamrick (1996a) found significant differences in the articular curvature of the distal radius of lorises compared to other strepsirrhine taxa (Figure 2A–C). Hamrick (1996a) also reported a morphocline in the shape of the midcarpal joint, with pronograde quadrupeds having a flatter joint (Figure 2A), vertical clingers having a more curved joint (Figure 2B), and slow climbers having the most tightly curved joint (Figure 2C). Not only does the shape curvature of the proximal and midcarpal joints of lorises resemble that of more suspensory anthropoid primates, the ulnotriquetral contact is reduced in a manner analogous to that observed in hominoids (Cartmill and Milton 1977; Mivart 1867; Rose 1993; Sarmiento 1988).

Diversity in carpal morphology among strepsirrhines encompasses most of the variation seen in the entire primate order, and some of this variation has been linked to differences in hand use during positional behavior. Still, few data exist in living strepsirrhines and other primates on how the carpals move and interact with each other during specific movements of the hand. For example, Cartmill and Milton (1977) suggested that the hominoid‐like specializations of the proximal carpal joint of lorises could serve to increase ulnar deviation and supination of the hand but noted the lack of empirical evidence to support this contention and the need for testing in anesthetized animals. To this end, this comparative study presents quantitative data on the degree of ulnar deviation of the hand and differences in carpal bone kinematics using single‐plane radiographic methods for a living sample of eight strepsirrhine species under anesthesia representing all major locomotor groups (i.e., pronograde quadrupeds, vertical clingers, and slow climbers). Ulnar deviation was selected because it represents a fundamental movement of the hand during positional behavior in primates and other arboreal mammals. Ulnar deviation also provides the clearest radiographic view of individual carpal bone kinematics during hand motion. In spite of considerable technological advancements in the study of carpal kinematics in primates using 3D computed‐tomography radiography (Orr 2016; Orr et al. 2010, 2023; van Leewen et al. 2019, 2022), more traditional, single‐plane radiographic methods remain valuable tools to investigate wrist function in primates and other arboreal mammals (Daver et al. 2012; Jouffroy and Medina 2002; Mendel 1979; Schreiber 1934; Yalden 1972; Ziemer 1978), especially if using living animals. As emphasized by Kivell (2016), integrating comparative and in vivo studies is crucial to improve our understanding of wrist function in living primates, particularly the understudied strepsirrhines, in order to interpret fossil carpal bones.

## 2 Materials and Methods

All strepsirrhines used were housed at the Duke Lemur Center (Durham, NC). Eight strepsirrhine primate species from major locomotor categories relevant to this study were examined (see Table 1): four pronograde quadrupeds ( *Cheirogaleus medius* , *Eulemur fulvus, Lemur catta*, and *Varecia variegata* ), one pronograde quadruped with some commitment to clinging ( *Hapalemur griseus* ), one committed vertical clinger ( *Propithecus coquereli* ), and two slow climbers ( *Loris tardigradus* and *Nycticebus coucang* ). Three individuals per species (four for *Nycticebus*) were radiographed for a total of 25 individuals. All subjects used were adults with full fusion of the distal radial epiphysis and in good health with no obvious pathology on the hands.

**TABLE 1:** Duke Lemur Center (DLC) study sample with body masses and hand ulnar deviation angles (°).

Taxon (DLC #)	Body mass (g)	Ulnar deviation angle (°)
Pronograde quadrupeds		
*Cheirogaleus medius* (DLC 3621m)	295	39
*Cheirogaleus medius* (DLC 3619f)	285	41
*Cheirogaleus medius* (DLC 1662f)	215	49
*Eulemur fulvus* (DLC 5529m)	2940	31
*Eulemur fulvus* (DLC 6225m)	2270	44
*Eulemur fulvus* (DLC 6306m)	2250	47
*Hapalemur griseus* (DLC 1366m)	1020	36
*Hapalemur griseus* (DLC 1369f)	1120	46.5
*Hapalemur griseus* (DLC 1367f)	900	50
*Lemur catta* (DLC 6143m)	3280	32
*Lemur catta* (DLC 6229f)	2720	33
*Lemur catta* (DLC 6268m)	2470	38
*Varecia variegata* (DLC 5587f)	3590	40
*Varecia variegata* (DLC 6495f)	3230	41
*Varecia variegata* (DLC 6494f)	3240	48
Vertical clinger		
*Propithecus coquereli* (DLC 6610m)	3680	49
*Propithecus coquereli* (DLC 6723f)	4370	55
*Propithecus coquereli* (DLC 6727f)	4770	58
Slow climbers		
*Loris tardigradus* (DLC 1981m)	190	87
*Loris tardigradus* (DLC 2914f)	175	90
*Loris tardigradus* (DLC 1921f)	170	95
*Nycticebus coucang* (DLC 1998f)	1280	64
*Nycticebus coucang* (DLC 1959m)	—	72
*Nycticebus coucang* (DLC 1971f)	975	77
*Nycticebus coucang* (DLC 1960m)	970	93

Abbreviations: f = female; m = male.

Animal subjects were anesthetized using an isoflurane mask (5% induction; then 2% maintenance) as part of their routine yearly physical examination. The right hand and distal forearm of each animal were radiographed using an Innovet V125 x‐ray system (Summit Industries Inc., Chicago, IL). High‐contrast Agfa Radiomat films (400‐speed rare‐earth 10 x 12 in.) placed in cassettes with green rare‐earth intensifying screens (MCI Optonix LLC, Washington, NJ) were shot at 10 mAs and between 46 and 49 kVp depending on the size of the animal. Radiographs of the hand and distal forearm with the palm and digits facing down onto the cassette were taken in a *neutral position* (i.e., pronated hand with its dorsal aspect in line with the dorsal aspect of the forearm and no lateral deviation) and *maximum ulnar deviation position* (i.e., pronated hand with its dorsal aspect in line with the dorsal aspect of the forearm and ulnarly deviated until no motion was possible). In each anatomical position, the hand and forearm of the animal were taped down to restrict any movement during the procedure. Vital signs were checked periodically during the procedures, which lasted 20 to 25 min for each animal. These procedures were approved by the Duke University Institutional Animal Care and Use Committee (Registry #206‐02‐07).

**FIGURE 3:**
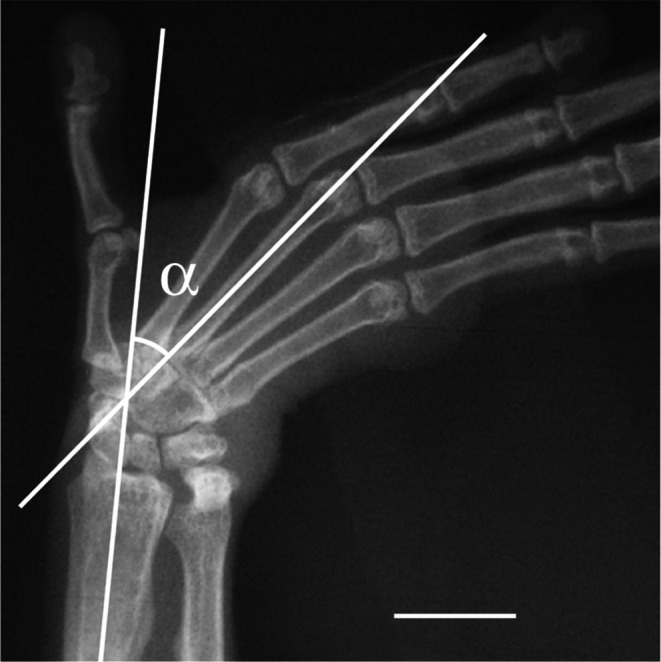
Radiograph of the hand in maximum ulnar deviation for *Lemur catta* (Scale bar = 1 cm). Two lines superimposed onto the image were drawn to measure the maximum ulnar deviation angle (α) as indicated.

All radiographs were digitally photographed and imported as JPEG files into a computer. Maximum ulnar deviation of the hand was measured with ProAnalyst v.1.5 motion analysis software (Xcitex Inc., Cambridge, MA) by drawing lines onto the digital radiograph parallel to the long axes of the distal half of the radius and third metacarpal/capitate, which are both straight axes in all eight strepsirrhine species examined (Figure 3). The resulting angle was computed by the software and rounded up to the nearest 0.5° (°). Differences in ulnar deviation angulation were tested among the strepsirrhine sample with a Kruskal‐Wallis test (i.e., nonparametric equivalent of a single classification ANOVA; see Sokal and Rohlf 1995) using the JMP v.5.1 statistical software (SAS Institute Inc., Cary, NC). Positioning of some of the carpal bones between neutral and maximum ulnar deviation postures of the hand was visually inspected for each radiograph to verify pattern consistency within species and within locomotor categories. Major differences in carpal positioning between the two hand postures were then compared qualitatively between locomotor categories. They include: (1) scaphoid and lunate displacement relative to the distal radius joint surface, (2) triquetrum‐pisiform complex displacement relative to the distal ulnar joint surface, (3) capitate and hamate displacement at the midcarpal joint, and (4) presence/absence of an ulnotriquetral contact (see Table 2 for summary).

**TABLE 2:** Summary of carpal joint motions and features observed during maximal ulnar deviation in the sample of strepsirrhine primates.

Carpal joint variable	Pronograde quadrupeds	Vertical clinger	Slow climbers
Displacement the of scaphoid and lunate relative to the distal radius joint surface	Minimal	Some radial translation of the scaphoid Space present between the scaphoid and lunate	Extensive translation of the scaphoid and lunate Lack of contact of the scaphoid with the radius Broad lunate contact with the radius
Displacement of the capitate and hamate at the midcarpal joint	Some translation	Some translation and rotation	Extensive translation and rotation
Displacement of the triquetrum‐pisiform complex relative to the distal ulnar joint surface	Minimal with some movement of the pisiform	Minimal with some movement of the pisiform	Proximal and radial displacement of the triquetrum‐pisiform complex
Ulnotriquetral contact	Present	Present	Absent

## 3 Results

**FIGURE 4:**
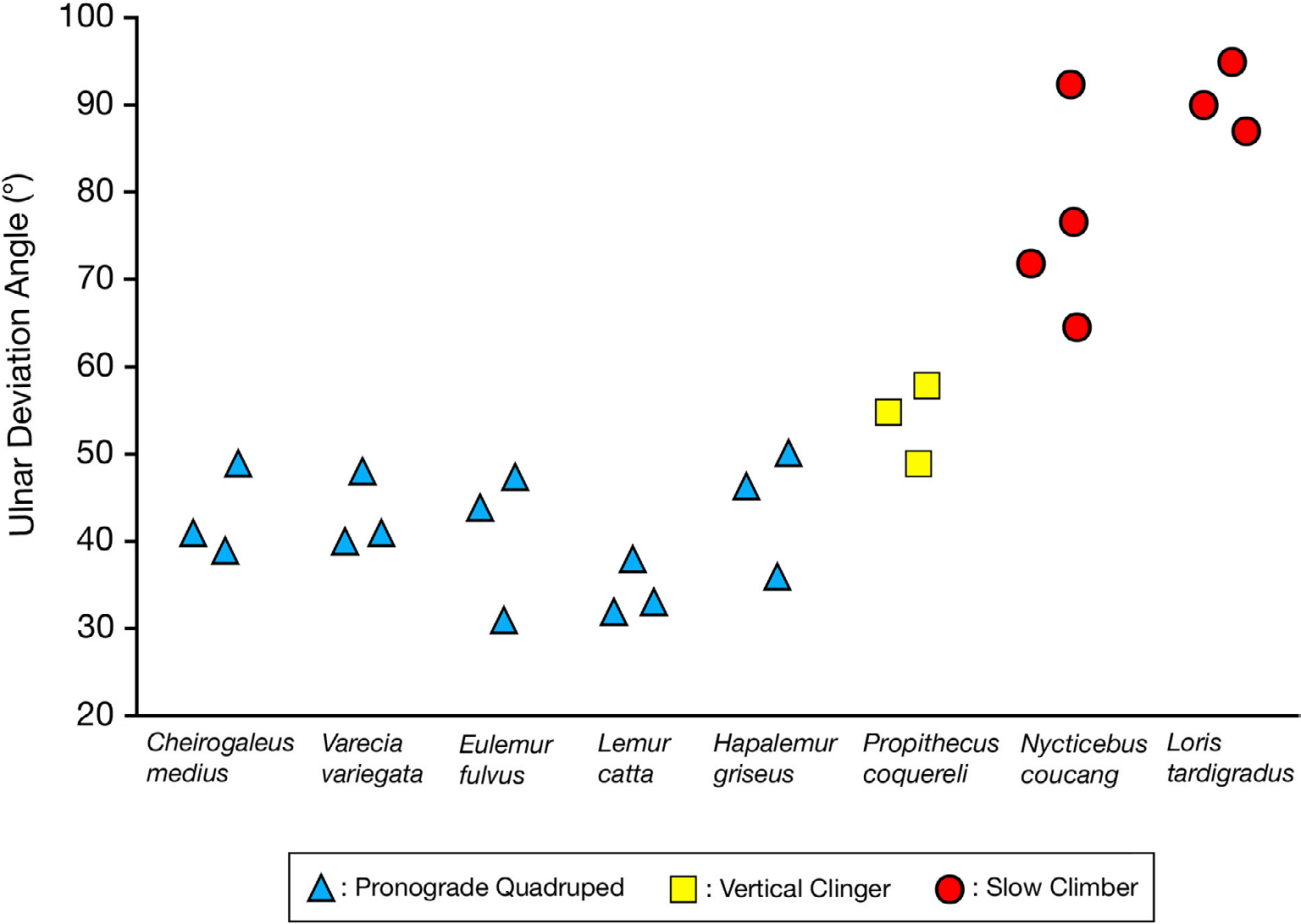
Angle values (°) for maximum ulnar deviation of the hand in eight strepsirrhine species. Note the steady increase in angle values from pronograde quadrupeds (blue triangles), vertical clinger (yellow squares), to slow climbers (red circles).

Differences in ulnar deviation abilities of the hand closely parallel differences in carpal joint architecture and locomotor behavior documented for strepsirrhine primates (Table 1; Figure 4). Within the lemurs, lower degrees of ulnar deviation are found in pronograde quadrupeds (with the lowest values in the more terrestrial *Lemur catta* ) and higher degrees in the vertical clinging *Propithecus* (Table 1; Figure 4), although those differences are not significant (χ^2^ = 9.61, df = 5, *p* > 0.05). The range of values for the vertical clinging *Propithecus* exceeds that of all pronograde quadrupeds, including *Hapalemur*. Among the pronograde quadrupeds, the highest degree of ulnar deviation is found in one individual of *Hapalemur*. This is most likely not coincidental as the locomotor repertoire of *Hapalemur* involves more vertical clinging behavior compared to other lemurids (Napier and Walker 1967; Walker 1979). The locomotor repertoire of *Hapalemur* has been described as intermediate between *Eulemur fulvus* , a pronograde quadruped, and vertical clinging indriids such as *Propithecus* (Petter et al. 1977; Fleagle and Anapol 1992). Slow climbing lorises are characterized by more extreme ulnar deviation of the hand, with angle values that do not overlap with any of the lemurs, whether pronograde quadrupeds or the vertical clinger (Table 1; Figure 4). Those differences are highly significant (χ^2^ = 19.77, df = 7, *p* < 0.01).

**FIGURE 5:**
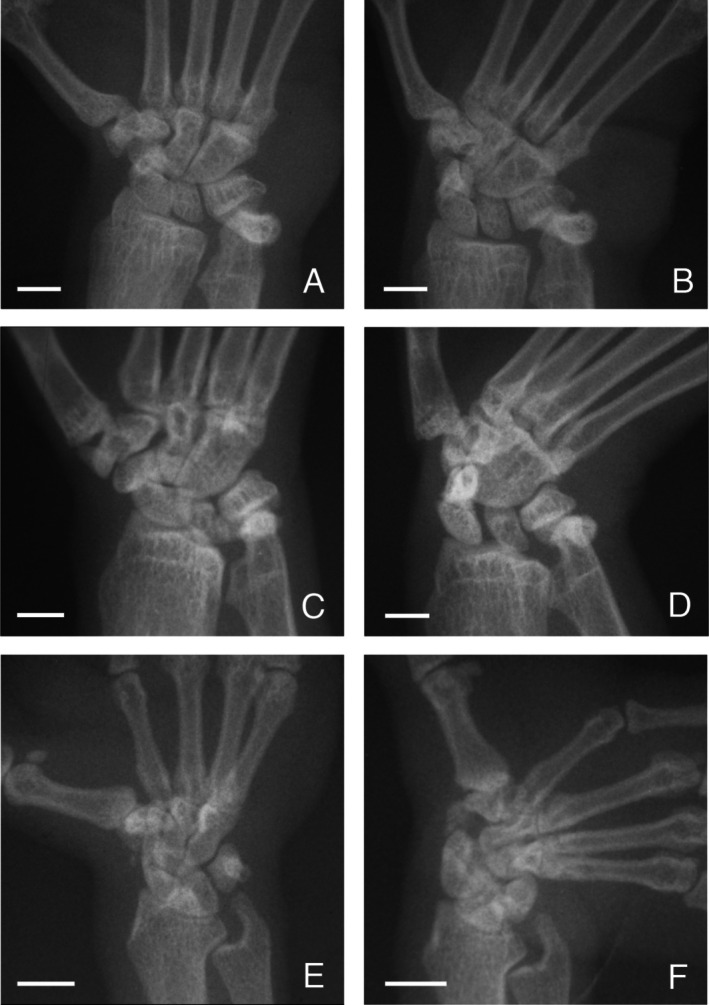
Radiographs of the hand in neutral position (left) and maximum ulnar deviation position (right) in a pronograde quadruped ( *Varecia variegata* ) (A, B), vertical clinger ( *Propithecus coquereli* ) (C, D), and slow climber ( *Nycticebus coucang* ) (E, F) (Scale bars = 1 cm).

**FIGURE 6:**
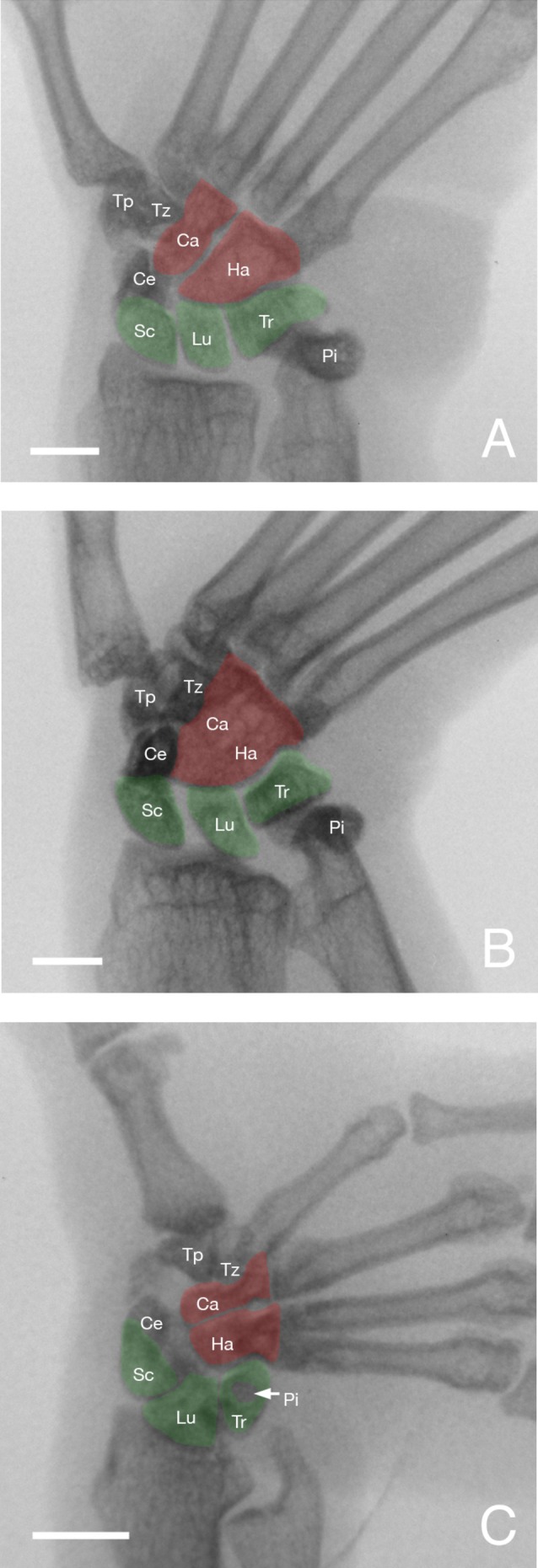
Inverted radiographs of the hand in maximum ulnar deviation position in *Varecia variegata* (A), *Propithecus coquereli* (B), and *Nycticebus coucang* (C) (Scale bars = 1 cm). Ca: capitate, Ce: os centrale, Ha: hamate, Lu: lunate, Pi: pisiform, Sc: scaphoid, Tr: triquetrum. Note the relative position between taxa of the carpals of the proximal row (in green) and distal row (red) involved in the proximal carpal and midcarpal joints. Extreme ulnar deviation of the hand observed in *Nycticebus* is accompanied by ulnar rotation and translation of the hamate onto the triquetrum, radial translation of the scaphoid and lunate onto the distal articular surface of the radius, and lack of an apparent ulnotriquetral contact.

Two similar articular mechanisms are observed during ulnar deviation of the hand in strepsirrhine primates: (1) at the proximal carpal (i.e., antebrachiocarpal) joint, radial translation (i.e., sliding; see appendix A in MacConaill and Basmajian 1969) of the proximal scaphoid and lunate takes place against the distal surface of the radius; (2) at the midcarpal joint, a combination of rotation (i.e., roll; see appendix A in MacConaill and Basmajian 1969) and radial translation of the proximal capitate and hamate takes place against the distal surface of the scaphoid/os centrale, lunate, and triquetrum (Table 2; Figures 5 and 6). That said, movements of the carpals at these joints vary substantially, particularly between the lemurs and lorises, because of shape differences (e.g., degree of curvature and congruence between the conarticular or male and female surfaces of the joint; see MacConaill and Basmajian 1969), contact (or lack of) with neighboring bones, and other factors more difficult to assess (e.g., ligament laxity, muscle tendon elasticity, synovial fluid quantity and displacement during movement, etc.). Those same factors likely affected individual variation in the functional ability to ulnar deviate the hand (e.g., variation in ulnar deviation within *Nycticebus*).

In pronograde quadrupedal lemurs such as *Varecia*, the proximal carpals show little translation during ulnar deviation while the hamate slides for a short distance before resting obliquely (in what appears to be close‐packed position on the radiograph) against the distal triquetrum (Figures 5A,B and 6A). The joint contact between the ulnar styloid process, triquetrum, and pisiform shows little difference as hand positioning changes. Only the pisiform appears to move slightly distally during maximum ulnar deviation, perhaps indicating a close‐packed position of the tip of the ulnar styloid process inside the cup formed by the triquetrum and pisiform (Figure 5A,B; see also Hamrick 1996c). In all, movements of the capitate and hamate at the midcarpal joint appear to be most notable during ulnar deviation (Table 2).

In the vertical clinging *Propithecus*, the scaphoid and lunate show more translation (with a notable space developing between the bones) in maximum ulnar deviation compared to other lemurs; in that hand position, about half of the scaphoid projects away from the margin of the radius and does not contact its distal articular surface (Figures 5C,D and 6B). This radial translation of the scaphoid (with the space developing between it and the lunate) appears to allow the capitate and hamate to undergo some translation as they rotate against the distal articular surface of the more proximal carpals. In maximum ulnar deviation, the articular surfaces of the hamate and triquetrum are highly congruent. The ulno‐distal corner of the triquetrum fits tightly in a notch on the ulnar side of the hamate that is more notable than in other lemurs (Figures 5D and 6B). Like *Varecia* and other quadrupedal lemurs, the components of the ulnocarpal joint show little movement during ulnar deviation. This is not the case for the inferior (distal) radioulnar joint. The gap seen between the articular surfaces of the distal radius and ulna disappears with the hand in maximum ulnar deviation (Figure 5C,D). Compared to pronograde quadrupeds, ulnar deviation of the hand in *Propithecus* involves not only movement of the capitate and hamate at the midcarpal joint, but also some movement of the scaphoid and lunate at the proximal carpal joint (Table 2).

The hand of slow climbing lorises displays a much broader range of motion as the hand undergoes ulnar deviation. Associated with this enhanced range of motion is even more notable displacement of many of the carpal bones compared to pronograde quadrupedal and vertical clinging lemurs. At the proximal carpal joint, radial translation and ulnar rotation both take place simultaneously. Translation of the scaphoid is so extreme that its articular contact with the distal radius is lost when the hand reaches maximum ulnar deviation (Figures 5E,F and 6C). Concurrently, the broad and convex surface of the proximal lunate slides and occupies the entire concave and highly congruent articular surface of the distal radius. As a result, the lunate is the only carpal contacting the radius when the hand of lorises achieves maximum ulnar deviation (Figure 6C). The triquetrum (and the diminutive pisiform) moves proximally and radially during ulnar deviation, and the absence of contact (real or apparent) with the reduced styloid process of the ulna clearly contributes to such movement of the triquetrum (Figures 5E,F and 6C). At the midcarpal joint, the ball formed by the adjacent articular surfaces of the capitate and hamate slides and rotates against the socket formed by the more proximal carpals (i.e., scaphoid/os centrale, lunate, and triquetrum; Figure 6C). Like *Propithecus*, the ulno‐distal corner of the triquetrum forms a close contact with a notch on the ulnar side of the hamate (Figure 6C). In contrast to pronograde quadrupedal and vertical clinging lemurs, ulnar deviation of the hand in slow climbing lorises is accompanied by equally notable movements of the carpals at both proximal carpal and midcarpal joints (Table 2).

## 4 Discussion

There is a clear relationship between the morphology of the proximal carpal and midcarpal joints and hand mobility in strepsirrhine primates. The observed differences in ulnar deviation angles follow closely differences in hand postures during positional behavior (Figure 1) and curvature of the proximal and midcarpal carpal joints for strepsirrhines (Hamrick 1996a, 1996c; Figure 2A–C). The more curved the joint surfaces are (concave or convex), the more movement they appear to promote (see Hamrick 1996a). As such, a more concave and curved distal articular surface of the radius at the proximal carpal joint and convex articular surface of the hamate forming a steeper slope with the distal articular surface of the triquetrum at the midcarpal joint are associated with more pronounced ulnar deviation of the hand. In all strepsirrhines, ulnar deviation of the hand involves varying degrees of rotation (roll) accompanied by some translation (sliding) of the capitate and hamate at the midcarpal joint. In lorises, notable radial translation of the scaphoid and lunate at the proximal carpal joint also takes place during ulnar deviation. Not surprisingly, lorises achieve degrees of ulnar deviation that are significantly greater than those of lemurs (both pronograde quadrupeds and vertical clingers). The ability of those wrist joints to achieve greater degrees of translation and rotation in lorises is highly dependent on the geometry of the joint surfaces involved in the proximal and midcarpal carpal, in particular the distal radius, proximal scaphoid and lunate, and proximal capitate and hamate. Moreover, the diminutive ulnar styloid process of lorises allows for movement of the triquetrum during ulnar deviation, which in turn promotes more translation and/or rotation of adjacent carpals.

Moving of the hand in ulnar deviation using the carpal joint mechanisms described above is not only typical of lemurs and lorises, but also of other major primate groups, including humans (Jouffroy and Medina 2002; Orr et al. 2023; Sarmiento 1988; Schreiber 1934; Wood Jones 1942; Yalden 1972; Ziemer 1978). That said, great apes and humans appear to have more ulnar deviation taking place at the proximal carpal joint, whereas most other primates (strepsirrhines, monkeys, and hylobatids) rely on the midcarpal joint to achieve the same hand motion (Daver et al. 2012; Jouffroy and Medina 2002; Sarmiento 1988; Yalden 1972).

In *Varecia*, a pronograde quadruped, the position of the proximal carpals shows little differences between hand positions (Figure 5A,B). The scaphoid and lunate remain in contact with the distal radius, and further motion of these carpal bones is limited when the triquetrum and pisiform achieve a close‐packed position with the bulbous and prominent ulnar styloid process. Previous studies suggested that this ulnocarpal contact limits, but stabilizes, the hand in ulnar deviation (Beard and Godinot 1988; Beard et al. 1986; Hamrick 1996b, 1996c; Lemelin and Schmitt 1998; O'Connor 1975; Orr et al. 2023). In vivo data here confirm that during ulnar deviation, the ulnar styloid process and associated ligaments stabilize the triquetrum, which in turn restricts motion of the scaphoid and lunate at the proximal carpal joint. The end result is that ulnar deviation takes place primarily at the midcarpal joint in *Varecia* and other pronograde quadrupedal lemurs (Figures 5A,B and 6A). Like other quadrupedal primates, the orientation of the triquetrohamate facet is more proximal (i.e., radioulnar orientation), thus better adapted for weight bearing, but in turn limits ulnar deviation (Fleagle 1977; Fleagle and Meldrum 1988; Godinot and Beard 1991; Hamrick 1996b; Jenkins 1981; Kivell 2016; Lemelin et al. 2008; Orr et al. 2023). However, the surface of the midcarpal joint is more curved in pronograde quadrupedal strepsirrhines compared to that of pronograde quadrupedal anthropoids (Hamrick 1996c). Accordingly, the range of ulnar deviation observed in pronograde quadrupedal strepsirrhines (between 31° and 50°; Table 1) is somewhat higher compared to pronograde quadrupedal monkeys (between 22° and 41°; Daver et al. 2012; Orr et al. 2023). Rhesus monkeys ( *Macaca mulatta* ) are a notable exception with an ulnar deviation median value of 54° (Orr et al. 2023). Overall, the relationship between joint curvature and ulnar deviation appears to hold up even within a locomotor category like pronograde quadrupedalism. It should be noted that within the strepsirrhine sample, the lowest values of ulnar deviation are found consistently in *Lemur catta* (Table 1; Figure 4), the most terrestrial lemur (Gebo 1987; Ward and Sussman 1979).

In *Propithecus*, a vertical clinger, the position of some of the proximal carpals changes between hand positions (Figure 5C,D). Like *Varecia*, the ulnocarpal contact is also broad and limits movement of the triquetrum and pisiform. In spite of that, some radial translation seems to take place for the scaphoid and lunate, and a space clearly develops between those carpals in ulnar deviation (Figures 5C,D and 6B). Since the transverse radiocarpal joint surface has similar curvature as pronograde quadrupeds (Hamrick 1996a, 1996b), sliding movement of the scaphoid and lunate during ulnar deviation could be the result of a looser joint capsule and associated ligaments. Like *Varecia* and other pronograde quadrupeds, ulnar deviation appears to take place mainly at the midcarpal joint. However, the more curved joint surfaces of the capitate and hamate (Hamrick 1996a, 1996b) combined with a triquetrohamate facet oriented more laterally (i.e., proximodistal orientation) tapering into a notch for the triquetrum, increase (not significantly however) the ulnar deviation abilities of the hand of *Propithecus* compared to more pronograde quadrupedal lemurs (Table 1; Figure 4).

In *Nycticebus*, a slow climber, the position of most bones of the proximal carpal and midcarpal joints undergo notable changes as the hand achieves higher degrees of ulnar deviation (Figure 5E,F). Unlike pronograde quadrupedal lemurs, ulnar deviation appears to take place at both proximal carpal and midcarpal joints. The much‐reduced ulnocarpal joint promotes radial displacement of the triquetrum, which in turn leads to radial translation of the scaphoid and lunate. The highly curved joint surface of the distal radius of lorises (Hamrick 1996a) clearly plays an important role in promoting radial translation at the proximal carpal joint during ulnar deviation (Figures 5E,F and 6C). It is worth noting that the longitudinal septum typical of all strepsirrhine primates, which divides the proximal carpal joint into radial and ulnar compartments and connects the lunate and triquetrum (see Kivell 2016), does not appear to restrict ulnar deviation in lorises. Equally important is the highly curved and convex joint surface of the hamate (Hamrick 1996a) allowing the midcarpal joint to translate and rotate (Figures 5E,F and 6C).

**FIGURE 7:**
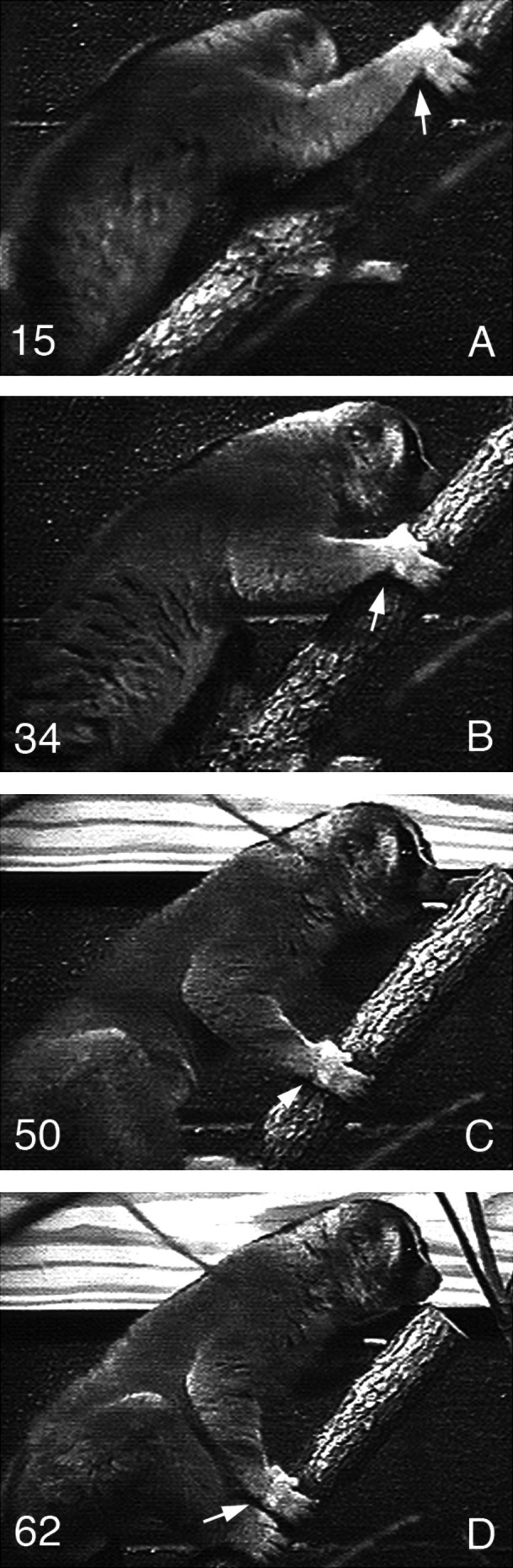
Sequential images of a *Nycticebus coucang* climbing up an oblique branch. The arrows indicate the extreme degree of ulnar deviation of the hand at touchdown (A) and the wide range of radioulnar deviation, flexion‐extension, and likely rotation of the hand at the wrist joints throughout the support phase (A–D). Note as well the position of the forearm parallel with the substrate at touchdown. Each image represents 1/30 s.

The proximal carpal and midcarpal joints of lorises are clearly adapted for enhanced mobility in multiaxial planes. Behaviorally, the more curved wrist joint surfaces of lorises are associated not only with wider ranges of radioulnar deviation, but also increased flexion‐extension and likely rotation of the hand during locomotion, particularly during ascent and vertical climbing (Figure 7A–D). In many ways, the wrist anatomy of slow climbing lorises combines the features found in the most suspensory primates and other mammals. For example, the joint surface of the distal radius of lorises extends as a shelf over the ulnar head (see Cartmill and Milton 1977), fulfilling the same functional role of the triangular articular disc found in hominoids. A similar extended shelf of the distal radius is also found in *Pongo* (Sarmiento 1988; Orr et al. 2023). As well, the lunate of slow climbing lorises is shaped like an acute trapezoid (i.e., the base is wider relative to the apex) (Beard and Godinot 1988; Etter 1978; Forster 1934; Godinot and Beard 1991). This wide base is the only articular contact with the highly curved and congruent distal articular surface of the radius when the hand is in maximal ulnar deviation (Figures 5F and 6C). This broad joint surface of the lunate not only promotes radial translation, but also likely provides stability and resistance to compressive loads when the hand is in maximal ulnar deviation (Hamrick 1996a; Orr et al. 2023). The same morphology typifies the lunate of *Pongo* (Jenkins and Fleagle 1975; Orr et al. 2023; Sarmiento 1988) and two‐toed sloths (Mendel 1979; Yalden 1972), both of which can achieve higher degrees of ulnar deviation compared to many quadrupedal primates, including African apes (Tuttle 1969a; Orr et al. 2023). Beard and Godinot (1988) noted a similar shape for the lunate of *Adapis*, although not as wide as in lorises, suggesting enhanced ulnar deviation abilities for the hand of this Eocene primate compared to *Smilodectes*.

The midcarpal joint of slow climbing lorises comprises a capitate and hamate that together are shaped like a bulbous knob with highly curved joint surfaces that fit together with the more proximal carpals shaped like a cup. This morphology resembles that of suspensory primates like *Ateles* and *Hylobates*, both of which are characterized by highly mobile midcarpal joints that rotate (i.e., pronation/supination) during bimanual locomotion (Jenkins 1981; Jenkins and Fleagle 1975). Because of its shape, it is also likely that the midcarpal joint of lorises is capable of rotation, in addition to the ulnar deviation demonstrated in this study.

It is noteworthy that primates that emphasize slow climbing in their locomotor repertoires, lorises and *Pongo* mainly, possess several convergent carpal features such as an extended radial shelf combined with a broad proximal lunate and a globular capitate head. The basic mechanism of ulnar deviation appears similar in these primates, with the lunate assuming a broad contact with the distal radius as it translates/rotates, and the capitate rotating at the midcarpal joint (Orr et al. 2023). In *Pongo*, the slender and derived triquetrum is positioned more distally next to the hamate and carries the pisiform away from the ulnar styloid process during ulnar deviation of the hand (Orr et al. 2023). In lorises, the triquetrum and small pisiform move together distally away from the ulnar styloid process as hand ulnar deviation takes place.

Like hominoids, lorises have a reduced ulnar styloid process. Much of the discussion about the evolution of the primate wrist morphology revolves around the functional and phylogenetic significance of the reduction of the ulnar styloid process in Hominoidea (Cartmill and Milton 1977; Conroy and Fleagle 1972; Lewis 1969, 1974, 1985, 1989; O'Connor 1975; Mendel 1979; Orr et al. 2023; Sarmiento 1988; see also Kivell 2016 for a thorough review). Lewis (1969) considered the reduced ulnar styloid process as a prerequisite for “true” brachiation and used it as evidence that humans and apes shared a brachiating ancestry. In their seminal paper, Cartmill and Milton (1977) argued that the presence of a reduced ulnar process (along with a reduced pisiform) in lorises was evidence that deliberate climbing was the common locomotor behavior that led to the convergent evolution of this morphological feature in both lorises and hominoids. This contention was espoused by Mendel (1979) based on the reduced ulnar styloid process observed in slow climbing two‐toed sloths. In a rebuttal of this hypothesis, Lewis (1985: 449) argued that the reduction of the ulnar styloid process in lorises is “secondary to a strikingly derived type of midcarpal joint” capable of an extreme degree of ulnar deviation accompanied by pronation and extension.

In his original description, Lewis (1969) also pointed out that a reduced ulnar styloid process increases the ability of the forearm to achieve greater ranges of pronation and supination by freeing it from articulation with the ulnar‐sided carpals. Hominoids have distal (inferior) radioulnar joints with extensive and curved surfaces, with the very large ulnar facet also facing distal and articulating with the triangular articular disc (O'Connor 1975; Rose 1993; Sarmiento 1988). Such an arrangement likely promotes enhanced ranges of pronation and supination of the forearm. Interestingly, slowclimbing lorises also possess a relatively large and evenly curved ulnar facet at the inferior radioulnar joint compared to other strepsirrhines (Rose 1993). The shelf‐like extension of the distal radius not only provides a greater surface for the proximal carpals but also a wider surface for the ulnar head to articulate (see Cartmill and Milton 1977). This configuration is likely to afford a wider arc of motion for forearm pronation and supination in lorises compared to other strepsirrhines, which should be expected in primates with frequent overhead reaching and grasping. Recruitment of forearm supinators to reach and grasp the underside of a vertical support with the hand during climbing is a vital behavior in hominoids (Stern and Larson 2001). This is most likely the case in slow climbing lorises as well.

Recent evidence from an anthropoid sample suggests that reduction of the ulnar styloid process is not linked directly with ulnar deviation enhancement, but rather due to a stress reduction on the ulnar side of the wrist or a by‐product of wrist adaptations emphasizing supination (Orr et al. 2023). Among strepsirrhines, greater ulnar deviation of the hand in lorises covaries with wrist adaptations that include a reduced ulnar styloid process as shown by these comparative results. All that said, the reduction of the ulnar styloid process may serve different roles in primates and should be viewed as one of many parts of a functional complex responsible for increased ranges of motion of the hand in primates.

### 4.1 Study Limitations

Because of the small size of the carpals of strepsirrhine primates and resolution of the radiographs, homologous landmarks could not be tracked accurately between hand positions. As a result, the individual contribution of the proximal carpal joint vs. midcarpal joint in ulnar deviation of the hand could not be quantified in this study. Despite this limitation, the contribution of those wrist joints during ulnar deviation of the hand between pronograde quadrupedal, vertical clinging, and slow climbing strepsirrhines could still be visualized (Figures 5 and 6) and described qualitatively.

Although ulnar deviation is a fundamental movement of the primate hand during locomotion and posture, so are the rotational abilities in pronation and supination, as discussed above. As much as hand rotational movement data would have been highly desirable to collect, measuring such motions and imaging associated carpal bone positioning are technically challenging. Superimposition of individual carpal outlines occurs when the radioulnar sides of the hand are radiographed. This problem is compounded when imaging smaller primates like a slow loris. Moreover, working with live animal subjects under anesthesia imposes major limits on the number of views that can be radiographed per subject in a given session. Future studies of wrist function in strepsirrhine primates could consider an ex vivo approach to joint mobility using X‐Ray Reconstruction of Moving Morphology (XROMM) technology (see Manafzadeh 2020 for details).

## 5 Conclusions

Differences in wrist joint morphology are functionally linked to greater ranges of ulnar deviation observed in strepsirrhine primates during positional behavior (Figure 1). Steeper curvature of the distal radius in lorises (Hamrick 1996a; Figure 2A–C) allows for radial translation of the proximal carpals, particularly the broad lunate, during ulnar deviation of the hand. Moreover, the morphocline observed in the relative curvature of the midcarpal joint among strepsirrhine primates of different locomotor categories (from pronograde quadrupeds, vertical clingers, to slow climbers; Hamrick 1996a; Figure 2A–C) closely matches degrees of ulnar deviation of the hand observed on anesthetized subjects (Table 1; Figure 4). The more curved midcarpal joint surfaces of lorises allow for rotation and translation of the capitate and hamate during ulnar deviation of the hand. The broad and prominent ulnotriquetral contact observed in pronograde quadrupeds like *Varecia* and vertical clinging *Propithecus* clearly limits but also stabilizes the hand in ulnar deviation. In contrast, the reduced ulnar styloid process and lack of contact (real or apparent) with the triquetrum are part of an overall functional complex emphasizing carpal mobility in lorises, including greater ulnar deviation of the hand. These experimental data, which Cartmill and Milton (1977) advocated for nearly 50 years ago, clarify form‐function relationships in the wrist region of extant primates. Such functional relationships are critical to reconstruct hand use during positional behavior and the overall adaptive profile of extinct primates.

## Author Contributions

**Pierre Lemelin:** conceptualization (lead), data curation (lead), formal analysis (lead), funding acquisition (lead), investigation (lead), methodology (lead), project administration (lead), resources (lead), software (lead), supervision (lead), validation (lead), visualization (lead), writing – original draft (lead), writing – review and editing (lead).

## Conflicts of Interest

The author declares no conflicts of interest.

## Data Availability

The data supporting the findings of this study are available upon reasonable request.
